# The effect of PrP^Sc^ accumulation on inflammatory gene expression within sheep peripheral lymphoid tissue

**DOI:** 10.1016/j.vetmic.2015.10.013

**Published:** 2015-12-31

**Authors:** Anton G. Gossner, John Hopkins

**Affiliations:** The Roslin Institute & R(D)SVS, University of Edinburgh, Easter Bush, Midlothian EH25 9RG, UK

**Keywords:** Sheep, Scrapie, Lymph node, Inflammation, Transcriptome

## Abstract

Accumulation of the misfolded prion protein, PrP^Sc^ in the central nervous system (CNS) is strongly linked to progressive neurodegenerative disease. For many transmissible spongiform encephalopathies (TSEs), peripheral lymphoid tissue is an important site of PrP^Sc^ amplification but without gross immunological consequence. Susceptible VRQ homozygous New Zealand Cheviot sheep were infected with SSBP/1 scrapie by inoculation in the drainage area of the prescapular lymph nodes. The earliest time that PrP^Sc^ was consistently detected by immunohistology in these nodes was D50 post infection. This transcriptomic study of lymph node taken before (D10) and after (D50) the detection of PrP^Sc^, aimed to identify the genes and physiological pathways affected by disease progression within the nodes as assessed by PrP^Sc^ detection. Affymetrix Ovine Gene arrays identified 75 and 80 genes as differentially-expressed at D10 and D50, respectively, in comparison with control sheep inoculated with uninfected brain homogenate. Approximately 70% of these were repressed at each time point. RT-qPCR analysis of seven genes showed statistically significant correlation with the array data, although the results for *IL1RN* and *TGIF* were different between the two technologies. The ingenuity pathway analysis (IPA) and general low level of repression of gene expression in lymphoid tissue, including many inflammatory genes, contrasts with the pro-inflammatory and pro-apoptotic events that occur within the CNS at equivalent stages of disease progression as assessed by PrP^Sc^ accumulation.

## Highlights

• Arrays quantified gene expression in peripheral LNs during sheep scrapie.
• Disease progression associated with alterations of inflammatory gene expression.
• Lymph node response contrasts with response of CNS.
• Step changes to gene expression after the detection of PrP^Sc^ in peripheral LNs.

## 1 Introduction

Sheep scrapie is a transmissible spongiform encephalopathy (TSE), a group of fatal neurodegenerative diseases of the central nervous system (CNS). A key feature of TSEs is the conversion of the host-encoded prion protein PrP^C^ to disease-associated PrP^Sc^ (Prusiner, 1982); the replication of pathological PrP^Sc^ from physiological PrP^C^ is a critical component of the disease (Prusiner et al., 1999). The essential role of PrP^C^ in TSE disease is confirmed by the resistance of PrP^null^ mice to disease (Bueler et al., 1993); by the reciprocal relationship of PrP gene (*PRNP*) copy number and incubation period (Bueler et al., 1993, Manson et al., 1994), and by the fact that resistance to sheep scrapie is influenced by polymorphisms of *PRNP* at codons 136 (V or A), 154 (R or H) and 171 (R or Q) (Goldmann et al., 1994). With SSBP/1 scrapie in Cheviot sheep, VRQ homozygotes have the shortest incubation period (Houston et al., 2002).

The CNS is the major target organ for TSE disease and neurodegeneration is associated with the accumulation of PrP^Sc^ within neurons (Mallucci et al., 2003). Many TSE agents, including natural sheep scrapie, are associated with replication of infectivity in peripheral lymphoid tissue prior to the invasion of the CNS (Mabbott and Bruce, 2003). PrP^Sc^ replicates in follicular dendritic cells (FDC) in spleen and lymph node germinal centres (Jeffrey et al., 2000, McCulloch et al., 2011) and interference of this prolongs the incubation period. However, in contrast to neurons, PrP^Sc^ replication by FDC does not lead to their degeneration or the inhibition of gross immunological functions (Heikenwalder et al., 2005).

The effects of PrP^Sc^ accumulation on the CNS transcriptome has been investigated in several different species, including mice (Xiang et al., 2004), cattle (Almeida et al., 2011), sheep (Filali et al., 2012, Gossner and Hopkins, 2014) and humans (Tian et al., 2013) with the aim of identifying genes associated with TSE disease progression. Similar analysis of secondary lymphoid tissues is so far limited to two sheep studies; an investigation on mesenteric lymph node in natural scrapie (Filali et al., 2014) and our preliminary study (Gossner et al., 2011b) on SSBP/1 scrapie. The earliest time that PrP^Sc^ was consistently detected by immunohistochemistry was at 50 days post infection (D50), in the prescapular lymph node (PSLN) draining the site of scrapie inoculation; and microarray analysis of PSLN and spleen at D75 linked repression of inflammation with the accumulation of PrP^Sc^. This current study exploits the same model to compare, using the new Affymetrix Ovine Gene 1.1 ST whole-genome expression array and by RT-qPCR, the effects of scrapie infection on the transcriptome of the PSLN early after infection (D10) and after the immunohistochemical detection of PrP^Sc^ (D50). In this way we aim to identify how scrapie infection and/or PrP^Sc^ affect the molecular physiology of secondary lymphoid tissue; and to compare the events in this tissue to the CNS at equivalent stages of disease progression as assessed by PrP^Sc^ accumulation.

## 2 Materials and methods

### 2.1 Animals and experimental design

Animals, infections and tissues have been described in detail previously (Gossner et al., 2011a, Gossner et al., 2011b). Briefly, Cheviot sheep with *PRNP* homozygous genotype VRQ/VRQ (Houston et al., 2002) were inoculated subcutaneously in the drainage area of the PSLNs with either SSBP/1 brain homogenate (infected) or similarly prepared scrapie-negative brain homogenate; both brain homogenates contained PrP of both VRQ and ARQ genotypes. Three infected and two uninfected controls were killed at 10 days (D10) and 50 days (D50) post infection. Animal experiments were approved by BBSRC Institute for Animal Health Ethical Review Committee and conducted under an Animals (Scientific Procedures) Act 1986 Project Licence.

### 2.2 Sample collection and total RNA isolation

Tissues were removed post-mortem, dissected into blocks and submerged in RNA*later*^®^ (Ambion) incubated at 4 °C overnight and stored at −80 °C. Total RNA was isolated using the RiboPure™ RNA Purification Kit (Ambion, Huntingdon, UK) with DNase I digestion. RNA quality and integrity was assessed using the Agilent RNA 6000 Nano kit on the Agilent 2100 Bioanalyzer and quantified with a NanoDrop ND-1000 spectrophotometer.

### 2.3 RNA amplification and microarray hybridization

Transcriptome analysis was by Affymetrix Ovine Gene 1.1 ST arrays, which consist of 508,538 oligomers (25 mer) covering 22,047 genes. These are complementary to approximately 635 bases per gene and cover all exons of each annotated transcript of the Oar v2 sheep genome assembly. Sense-strand cDNA was generated from 0.5 μg of total RNA and subjected to two rounds of amplification using the Ambion^®^ WT Expression Kit. The cDNA was biotin labelled and fragmented using the Affymetrix GeneChip^®^ WT Terminal Labelling and Hybridization kit. Biotin-labelled fragments of cDNA (5.5 μg) were hybridized to the array plates using the appropriate Hyb-Wash-Scan protocol with reagents from the Affymetrix Gene Titan Hyb Wash Stain kit. After hybridization the plates were washed, stained and scanned by the Imaging Station of the GeneTitan System. The Affymetrix^®^ GeneChip^®^ Command Console^®^ Software (v3.0.1) was used to generate array images and the resulting Affymetrix intensity files (CEL files), along with the initial QC analysis.

### 2.4 Microarray data analysis

The CEL files were imported into Partek Genomics Suite ® software, version 6. 13.0213 (Copyright^©^ 2014; Partek Inc., St. Louis, MO, USA.) and data were analyzed at the gene-level using the mean expression of all exons of a gene. Background correction was performed using the robust multiarray average (RMA) algorithm, with quantile normalization, median polish probe summarization, and log_2_ probe transformation. Differentially-expressed genes were identified by analysis of variance (ANOVA), genes with a fold change >1.5 or <−1.5, and *p* value > 0.05 were retained. Hierarchical clustering was performed on significant genes, with the data normalized to a mean of zero and scaled to standard deviation of one using Partek. Significant genes were annotated based on similarity scores in BLASTN comparisons of Affymetrix Transcript cluster sequences against mRNA sequences in GenBank. The array data have been deposited in ArrayExpress database (www.ebi.ac.uk/arrayexpress) accession number E-MTAB-2327.

### 2.5 Functional enrichment and network analysis

Network analysis was performed through the use of QIAGEN’s Ingenuity Pathway Analysis (IPA^®^, QIAGEN Redwood City, www.qiagen.com/ingenuity) to increase confidence in the observations of differentially-expressed genes by correlation with biological pathways. This process also allowed the identification of putative key functional elements within the networks of differentially-expressed genes. The network interaction of the focused genes in the network is based on their connectivity in ingenuity knowledge base.

### 2.6 Quantitative real time RT-PCR (RT-qPCR)

Relative quantification of mRNA expression was conducted by RT-qPCR, using the FastStart Universal SYBR Master (Roche Applied Science) reaction mix. First strand cDNA synthesis was performed with 1 μg of total RNA using 0.5 μg oligo (dT)_15_ primer (Promega) and SuperScript^®^ II Reverse Transcriptase (Invitrogen) in a 20 μl final volume using the manufacturer’s recommended standard protocol.

RT-qPCR analysis was performed using a Rotor-Gene™ Q (Qiagen, Crawley, UK) with reactions prepared using a CAS-1200™ Precision Liquid Handling System (Qiagen). All reactions were performed in final volume of 15 μl and ‘no template’ controls were included for each primer pair. Gene specific primers sequences and PCR conditions for qPCR analysis are listed in Table S1. The amplification profile consisted of 10 min at 95 °C, followed by 40 cycles of gene-specific cycling conditions (Table S1), followed by a dissociation curve analysis. The cycle threshold value (Cq) was determined using the Rotor-Gene Q Software version 2.3.1 (build 49).

Agarose gel electrophoresis of amplicons confirmed a single product and sequence analysis was used to confirmed specificity of primer pairs. The linearity and efficiency of RT-qPCR amplification was determined for each primer pair (Table S1) using a standard curve generated by a dilution series of a pool of sample cDNAs.

### 2.7 RT-qPCR data analysis

The data analysis was based on a reaction efficiency-corrected modified comparative Cq method (ΔΔCq method) with gene expression normalized to the geometric mean of the reference genes SDHA and YWHAZ using the GenEx software version 5.3.4.157 (www.multid.se). To determine significant differences in gene expression between uninfected and SSBP/1 infected time points (D10 and D50), the log_2_ transformed data were compared using an unpaired *t*-test (2-tailed) within GenEx, *p* values < 0.05 were regarded as statistically significant. The Spearman’s rank correlation coefficient was performed using GraphPad Prism version 6.05 for Windows (GraphPad Software, La Jolla California USA, www.graphpad.com).

## 3 Results

### 3.1 Differential gene expression at D10 and D50

Array analysis identified 75 genes that showed significant difference, fold change ≥1.5 and adjusted *p* value ≤ 0.05, in the D10 vs. uninfected control (C) comparison; 23 were significantly increased (Table 1) and 52 were repressed (Table 2). At D50, 80 genes were differentially expressed, 18 were increased (Table 3) and 62 repressed (Table 4). No genes were common in the two comparisons, D10 vs. C and D50 vs. C; neither were there any significantly expressed genes (fold change ≥ 1.5 and *p* ≤ 0.05) in the direct comparison of the two infected groups, D10 vs. D50 (Table S2 and Table S3). The general repression of gene expression after infection is emphasized by heat maps for D10 (Fig. S1A) and D50 (Fig. S1B); in addition, they show the consistency of data from the individual sheep within the infected and control groups. The most up-regulated genes (and *p* ≤ 0.05) based on expression fold change included *IFI6* (+2.62 fold) and *ADM* (+2.30) at D10 and *GPR123* (+1.67) at D50. The most repressed included *SEPT5* (−2.31) and *Ovar-DYA* (−2.08) at D10; and *MOXD1* (−4.38), *CDH26* (−2.44) and *CAMP* (−2.43) at D50. The data were also analysed in relation to fold change without regard to *p* value (Table S4), and this showed that *HLA-DQA1* was the most differentially-expressed gene that increased 15.91 fold (*p* = 0.152) at D10 and 7.93 fold (*p* = 0.266) at D50. This list supports some of the data in Tables 1, S2 and S3, including a 1.67 fold repression of *MOXD1* (*p* = 0.302) at D10 (−4.38 fold at D50); a 1.54 fold increase in *IFI6* (*p* = 0.291) at D50 (+2.26 fold at D10) and a 2.54 repression of a second cathelicidin, *CATHL1B* also at D50. In addition, two inflammatory chemokines, *CCL26* and *CCL3L1* were repressed (−2.77 and −2.84 fold) and *AICDA* was 2.76 fold increased at D50.

### 3.2 Ingenuity pathway analysis

Ingenuity pathway analysis (IPA) was used to help characterize how differentially-expressed genes interact and affect the biological processes leading to the development of pathology in the SSBP/1 model of sheep scrapie in peripheral lymphoid tissue. The top Diseases and Disorders Bio Functions (Table 5) at D10 include ‘Gastrointestinal disease’ with 9 genes and the highest *p* value of 5.06 × 10^−4^, ‘Organismal injury and abnormalities (11 genes, *p* = 5.06 × 10^−4^) and ‘Neurological disease’ (16 genes, *p* value 5.06 × 10^−4^). At D50 the top Diseases and Disorders Bio Functions include ‘Neurological disease’ (8 genes *p* = 1.74 × 10^−4^), ‘Inflammatory response (5 genes, *p* = 3.24 × 10^−4^), ‘Immunological disease’ (13 genes, *p* = 1.97 × 10^−3^) and ‘Inflammatory disease’ (13 genes, *p* = 1.97 × 10^−3^).

### 3.3 RT-qPCR analysis

**Fig. 1:**
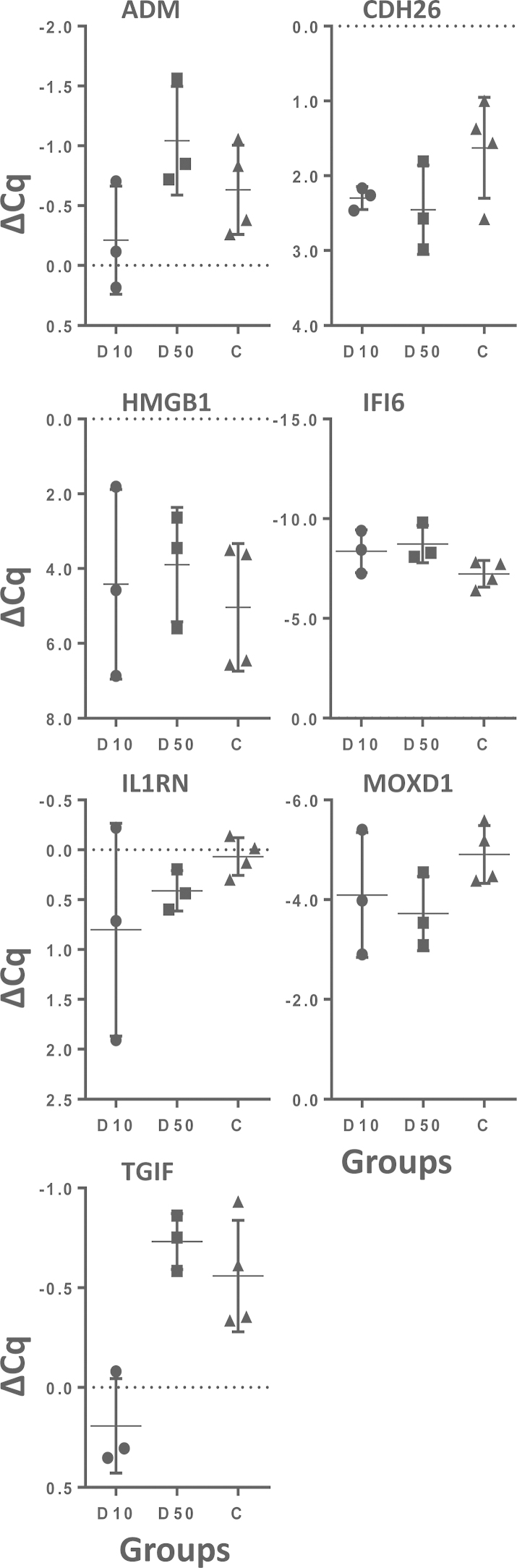
Relative expression of selected genes in the D10, D50 and uninfected control sample groups. RT-qPCR array results shown are the average ΔCq for the gene of interest (GOI) calculated using the following formula ΔCq = Cq^(GOI)^ − Cq^(geometricmeanofthereferencegenes)^. The mean for the animals in each group are shown with the error bars showing the standard deviation. Samples with the highest mRNA expression levels for a gene have the lowest ΔCq values. Circles represent the D10 samples (D10); squares represent the D50 samples (D50) and triangles represent the uninfected control samples (C).

Fold-change RT-qPCR of seven selected genes was used as independent validation of the microarray data. The relative expression levels of the genes in the D10, D50 and C groups is shown in Fig. 1 and the direct comparison of the fold changes obtained by Affymetrix arrays and by RT-qPCR is shown in Table 6. Spearman’s rank correlation analysis of the fold-change data shows correlation coefficients (*ρ*) of 0.6 (*p* = 0.17) for D10 and 0.94 (*p* = 0.005) for D50, and *ρ* = 0.6 (*p* = 0.01) for the combined D10/D50 data for all seven genes. RT-qPCR for *ADM*, *CDH26*, *HMGB1*, *IFI6* and *MOXD1* gave similar results to the microarray but *IL1RN* at D10, and *TGIF* at D50 gave different results with the two technologies.

## 4 Discussion

The current study builds on our previous projects (Gossner et al., 2011a, Gossner et al., 2011b, Gossner and Hopkins, 2014) using experimental SSBP/1 scrapie infection of VRQ homozygous New Zealand Cheviot sheep, and aims to identify the physiological processes triggered by SSBP/1 scrapie infection of peripheral lymph nodes at time points immediately before (D10) and after (D50) the detection of PrP^Sc^ by immunohistochemistry. These lymph nodes consist largely of lymphocytes that constantly traffic through the node; and there are no discernible changes to histology and/or cell content between the uninfected, D10 and D50 lymph nodes (Gossner et al., 2011b). However PrP^Sc^ accumulates and replicates in FDCs (McCulloch et al., 2011). Consequently the transcriptome signature of the node at any time point is likely to be principally determined by migrating lymphocytes responding to static, but PrP^Sc^-infected FDCs.

Inoculation of most infectious agents into the drainage area of a lymph node induces a reaction to that agent; principally the development of an adaptive immune response aimed at the elimination or control of that infection. For most antigens this response peaks at D5–D10 and resolves by D20 (Hall and Morris, 1965). Although scrapie infection does not seem to induce a specific immune response to the agent (a misfolded self-antigen) (Prusiner, 1982) or grossly affect the immunological function of the lymph node (Heikenwalder et al., 2005), 75 genes were significantly differentially-expressed by 10 days after SSBP/1 infection, in comparison to uninfected brain homogenate of the same *PRNP* genotypes. None of the 75 genes were significantly differentially-expressed at D50. Similarly, none of the 80 genes identified at D50 were changed significantly at D10. However there were several, e.g. *IFI6* at D10 and *MOXD1* at D50 that were differentially-expressed at the other time point but *p* > 0.05. This lack of obvious progression from D10 to D50 indicates a step change in lymph node physiology coincident with PrP^Sc^ accumulation and/or amplification similar to that seen with SSBP/1 scrapie in the CNS (Gossner and Hopkins, 2014).

Approximately 70% of differentially-expressed genes were decreased in relation to uninfected controls at both time points, indicating that general repression of transcription is an effect of scrapie infection even before the detection of PrP^Sc^ by immunohistochemistry. However, the levels of differential expression following scrapie infection were relatively modest; at D10 the maximum increase was only 2.62 fold and the maximum repression was 2.56 fold. At D50 the up-regulated genes varied only from 1.50 to 1.7 and the down-regulated genes from 1.51 to 4.48 fold. These data may indicate a general development of an anti-inflammatory response from D10 to D50, which confirms the conclusions from our preliminary study (Gossner et al., 2011b) on lymph node and spleen at the later D75 time point. The recently study (Filali et al., 2014) on mesenteric lymph node (MLN) of naturally-infected sheep also reported that the majority of differentially-expressed genes were repressed.

At D10 the top Bio-Functions were gastrointestinal and neurological disease; however some of the most significantly-increased genes have anti-inflammatory functions largely concerned with the blockade or inhibition of the major pro-inflammatory cytokines. Adrenomedullin (*ADM*, +2.30 fold) has a reciprocal relationship with IL-1β, IL-6 and TNFα (Koo et al., 2000); interleukin 1 receptor antagonist (*IL1RN*, +1.52 fold) specifically blocks IL-1α and IL-1β signalling (Arend et al., 1998) and immediate early response 3 (*IER3,* +1.6 fold) protect cells from TNFα and Fas-stimulated apoptosis (Wu, 2003). These inflammatory antagonists are usually released to regulate acute inflammation (Arend et al., 1998); however D10 was the earliest time point after SSBP/1 inoculation and we do not know if inflammation is an acute response to scrapie infection. In addition, some of the most significantly repressed genes are positively-associated with inflammation in other diseases. Leucine rich repeat containing 15 (*LRRC15*, -1.59 fold) is highly raised in caries-induced inflammation (Dolan et al., 2007); 5-hydroxytryptamine (serotonin) receptor 3A, ionotropic (*HTR3A*, -1.73 fold) increases the inflammatory effect of peripheral serotonin (Duerschmied et al., 2013) and neurofilament, light chain polypeptide (*NEFL*, -1.66 fold) is associated with inflammatory dysfunction driven by mitochondrial stress (Rossignol and Frye, 2014).

At D50 the top Bio-Functions were more obviously inflammation and immune response related, and major pro-inflammatory genes were repressed, including the anti-microbial cathelicidins *CAMP*, −2.33 fold (*CATHL3* in the sheep annotation) and *CATHL1B* (−2.54 but *p* > 0.05) and the inflammatory peptidase, cathepsin G (*CTSG*, −1.69 fold). *CATHL1B* was also found to be significantly repressed in MLN at the preclinical stage of natural scrapie (Filali et al., 2014). Several other genes linked with either oxidative or ER stress-associated inflammation are also down-regulated including transforming growth factor, homeobox 1 (*TGIF1*, −2.18) and dystrophin (*DMD*, −1.53). However, some adaptive immune-related genes were increased at *D50* including T cell differentiation protein (*MAL*, +1.58 fold) and high-mobility group box1 (*HMGB1*, +1.5 fold), which is a cofactor for RAG during VDJ recombination of immunoglobulin and T cell receptor genes and binds to TLR4 as an endogenous danger signal (Klune et al., 2008). Two other immunologically-relevant genes were also increased but with p values > 0.05. *HLA-DQA* (+15.91 fold at D10, +7.93 fold at D50) is expressed by macrophages, B cells and activated T cells (Hopkins et al., 1993); and activation-induced cytidine deaminase (*AICDA*, +2.78 fold) is involved in somatic hypermutation and therefore antigen-induced B cell maturation.

A significant body of work has shown that the progression of TSE diseases is associated with transition metal imbalance and a reduction in antioxidant activity (Thackray et al., 2002). PrP^C^ is a copper-binding molecule with anti-oxidant activity that protects cells from oxidative stress, and that low ferrous iron levels increase oxidative stress (Fernaeus et al., 2005). The reduction in expression of genes associated with transition metal metabolism was a consistent pattern at D50, and indicates an imbalance in copper and iron levels in sheep scrapie pathogenesis in lymphoid tissue. Ferritin, light chain (*FTL*, −1.55,) and lactotransferrin (*LTF*, −2.46,) play important roles in iron storage and homeostasis and are also increased during inflammation (Tran et al., 1997). Hephaestin (*HEPH*, −1.89,) is a multicopper oxidase that is involved in both copper and iron transport and homeostasis (Griffiths et al., 2005); and monoxygenase, DBH-like 1 (*MOXD1*, −4.38,) is copper-binding enzyme and part of the catecholamine pathway and reduced expression could lead to lower levels of dopamine and epinephrine, indicating possible consequent depression (Prigge et al., 2000). In addition, the chemokines *CCL26* (−2.77 fold) and *CCL3L1* (−2.84 fold), are major regulators of inflammation that promote eosinophil/basophil and lymphocyte/monocyte recruitment respectively (Griffith et al., 2014), were also repressed at D50 but with *p* values > 0.05.

There are two major conclusions from this study. Firstly, scrapie infection of the lymph node leads to the general down-regulation of genes and in particular is associated with the repression of inflammation at both D10 and D50. This contrasts with the effects of sheep scrapie-infection in the CNS, where studies (Filali et al., 2012, Gossner and Hopkins, 2014, Xiang et al., 2004) showed consistent increases in genes promoting complement fixation, inflammation and cell death/apoptosis as well as regulation of cell growth/cancer. Secondly, lymph nodes react to scrapie agent as early as 10 days after infection and this reaction is not related to the detectable accumulation of PrP^Sc^. An additional intriguing contrast in the response of the two tissues to SSBP/1 infection is the change in expression of olfactory receptor genes; in the CNS the majority are increased after infection while in peripheral lymphoid tissues only two are increased (at D10) and 19 are repressed (6 at day 10 and 13 at D50). These almost certainly represent the expression of non-functional pseudogenes within the lymph node (Zhang et al., 2007). However, as with the CNS in sheep scrapie, there seems to be no progression of events within the lymph node, from the early D10 time point to the later D50; which is consistent with gene expression changes in the MLN of preclinical and clinically-affected sheep (Filali et al., 2014). This implies that the alterations in gene expression reported in this current study are associated more with progression of disease rather than accumulation of PrP^Sc^.

## Competing interests

The authors declare that they have no competing interests.

## Figures and Tables

**Table 1:** Significantly increased differentially-expressed genes at D10.

Gene	Gene name	*p* value	FC
IFI6	Interferon, alpha-inducible protein 6	0.0422	2.62
ADM	Adrenomedullin	0.0482	2.30
ZNF347	Zinc finger protein 347	0.0282	2.04
XAF1	XIAP associated factor 1	0.0143	1.97
OR5S1P	Olfactory receptor, family 5, subfamily S, member 1 pseudogene	0.0225	1.87
GCSH	Glycine cleavage system protein H (aminomethyl carrier)	0.0496	1.85
MOGAT3	Monoacylglycerol *O*-acyltransferase 3	0.0309	1.78
ZBTB16	Zinc finger and BTB domain containing 16	0.0366	1.76
FKBP14	FK506 binding protein 14, 22 kDa	0.0495	1.73
NUDT15	Nudix (nucleoside diphosphate linked moiety X)-type motif 15	0.0353	1.73
S100A5	S100 calcium binding protein A5	0.0040	1.69
ESRP2	Epithelial splicing regulatory protein 2	0.0449	1.66
FAM171B	Family with sequence similarity 171, member B	0.0279	1.64
OR10H1	Olfactory receptor, family 10, subfamily H, member 1	0.0348	1.63
SERBP1	SERPINE1 mRNA binding protein 1	0.0265	1.62
IER3	Immediate early response 3	0.0151	1.60
RASGRF2	Ras protein-specific guanine nucleotide-releasing factor 2	0.0234	1.59
ZC3H12B	Zinc finger CCCH-type containing 12B	0.0049	1.58
GJA3	Gap junction protein, alpha 3, 46kDa	0.0122	1.57
IGFBPL1	Insulin-like growth factor binding protein-like 1	0.0218	1.57
DMXL2	Dmx-like 2	0.0107	1.56
MTERFD1	MTERF domain containing 1	0.0302	1.54
IL1RN	Interleukin 1 receptor antagonist	0.0337	1.52

Genes with fold change (FC) ≥ 1.5 fold and adjusted *p* value of ≤0.05.

**Table 2:** Significantly repressed differentially-expressed genes at D10.

Gene	Gene name	*p* value	FC
ASZ1	Ankyrin repeat, SAM and basic leucine zipper domain 1	0.0140	−2.56
SEPT5	Septin 5	0.0056	−2.31
Ovar-DYA	*O. aries* DNA for MHC class II DYA exon 3 (second domain)	0.0443	−2.08
RAB26	RAB26, member RAS oncogene family	0.0428	−2.01
ZNF391	Zinc finger protein 391	0.0347	−1.90
HNF4A	Hepatocyte nuclear factor 4, alpha	0.0421	−1.81
BRINP1	Bone morphogenetic protein/retinoic acid inducible neural-specific 1	0.0157	−1.78
LMF1	Lipase maturation factor 1	0.0379	−1.76
HTR3A	5-Hydroxytryptamine (serotonin) receptor 3A, ionotropic	0.0388	−1.73
CYP3A5	Cytochrome P450, family 3, subfamily A, polypeptide 5	0.0295	−1.73
MOGAT2	Monoacylglycerol *O*-acyltransferase 2	0.0078	−1.72
CLDN10	Claudin 10	0.0365	−1.71
ACOT4	Acyl-CoA thioesterase 4	0.0022	−1.71
OR8A1	Olfactory receptor, family 8, subfamily A, member 1	0.0259	−1.70
LOC101907318	LOC101907318 [*Bos taurus*]	0.0186	−1.68
DNAJB3	DnaJ (Hsp40) homolog, subfamily B, member 3	0.0003	−1.67
NEFL	Neurofilament, light polypeptide	0.0316	−1.66
RPL19	Ribosomal protein L19	0.0430	−1.66
FZD5	Frizzled class receptor 5	0.0493	−1.65
MAG	Myelin associated glycoprotein	0.0210	−1.64
HIST1H2AG	Histone cluster 1, H2ag	0.0122	−1.63
CXorf66	Chromosome X open reading frame 66	0.0240	−1.61
PRAMEF12	PRAME family member 12	0.0210	−1.61
TRMT2A	tRNA methyltransferase 2 homolog A (S. cerevisiae)	0.0487	−1.61
KRTAP9-1	Keratin associated protein 9-1	0.0119	−1.61
KRT35	Keratin 35	0.0484	−1.60
PHOSPHO1	Phosphatase, orphan 1	0.0057	−1.60

Gene	Gene name	*p* value	FC
OR10G9	Olfactory receptor, family 10, subfamily G, member 9	0.0362	−1.59
LRRC15	Leucine rich repeat containing 15	0.0059	−1.59
OR1P1	Olfactory receptor, family 1, subfamily P, member 1	0.0133	−1.58
PARD6B	par-6 family cell polarity regulator beta	0.0030	−1.58
KCNV1	Potassium channel, subfamily V, member 1	0.0036	−1.58
TMPRSS6	Transmembrane protease, serine 6	0.0367	−1.56
OR5B2	Olfactory receptor, family 5, subfamily B, member 2	0.0204	−1.56
NEGR1	Neuronal growth regulator 1	0.0325	−1.55
COBL	Cordon-bleu WH2 repeat protein	0.0235	−1.55
OR9K2	Olfactory receptor, family 9, subfamily K, member 2	0.0032	−1.55
ZNF398	Zinc finger protein 398	0.0380	−1.55
TRHDE	Thyrotropin-releasing hormone degrading enzyme	0.0119	−1.54
MKRN1	Makorin ring finger protein 1	0.0318	−1.54
GTPBP6	GTP binding protein 6 (putative)	0.0166	−1.54
ATCAY	Ataxia, cerebellar, Cayman type	0.0009	−1.54
ATP8A2	ATPase, aminophospholipid transporter, class I, type 8A, member 2	0.0068	−1.54
GPR68	G protein-coupled receptor 68	0.0462	−1.53
ZDHHC19	Zinc finger, DHHC-type containing 19	0.0022	−1.53
OR8B3	Olfactory receptor, family 8, subfamily B, member 3	0.0457	−1.53
PGA4	Pepsinogen 4, group I (pepsinogen A)	0.0001	−1.53
SLC7A9	Solute carrier family 7 (amino acid transporter light chain, bo,+ system), member 9	0.0213	−1.52
LIPN	Lipase, family member N	0.0218	−1.51
CCNI2	Cyclin I family, member 2	0.0057	−1.51
RPL10	Ribosomal protein L10	0.0007	−1.51
KHDRBS3	KH domain containing, RNA binding, signal transduction associated 3	0.0415	−1.50

Genes with fold change (FC) ≥1.5 fold and adjusted *p* value of ≤0.05.

**Table 3:** Significantly increased differentially-expressed genes at D50.

Gene	Gene name	*p* value	FC
TMEM116	Transmembrane protein 116	0.0342	1.74
HIST1H2AD	Histone cluster 1, H2ad	0.0075	1.73
LMO7	LIM domain 7	0.0287	1.72
ZNF471	Zinc finger protein 471	0.0050	1.70
GPR123	G protein-coupled receptor 123	0.0437	1.67
CYP4F8	Cytochrome P450, family 4, subfamily F, polypeptide 8	0.0147	1.67
ABCB6	ATP-binding cassette, sub-family B (MDR/TAP), member 6	0.0003	1.65
NAT1	*N*-acetyltransferase 1 (arylamine *N*-acetyltransferase)	0.0015	1.65
ZNF419	Zinc finger protein 419	0.0068	1.59
MRPL13	Mitochondrial ribosomal protein L13	0.0223	1.59
MAL	mal, T-cell differentiation protein	0.0487	1.58
MAGIX	MAGI family member, X-linked	0.0348	1.55
LYPLA2	Lysophospholipase II	0.0454	1.54
CYCS	Cytochrome c, somatic	0.0148	1.53
RPL29	Ribosomal protein L29	0.0095	1.52
ANKRD34A	Ankyrin repeat domain 34A	0.0379	1.51
HMGB1	High mobility group box 1	0.0279	1.50
VNN2	Vanin 2	0.0204	1.50

Genes with fold change (FC) ≥ 1.5 fold and adjusted p value of ≤ 0.05.

**Table 4:** Significantly repressed differentially-expressed genes at D50.

Gene	Gene name	*p* value	FC
MOXD1	Monooxygenase, DBH-like 1	0.0178	−4.38
LTF	Lactotransferrin	0.0400	−2.46
CDH26	Cadherin 26	0.0418	−2.44
CAMP	Cathelicidin antimicrobial peptide	0.0457	−2.43
CPNE4	Copine IV	0.0376	−2.33
OR5R1	Olfactory receptor, family 5, subfamily R, member 1	0.0214	−2.30
C11orf70	Chromosome 11 open reading frame 70	0.0452	−2.24
TGIF1	TGFB-induced factor homeobox 1	0.0310	−2.18
GABRQ	Gamma-aminobutyric acid (GABA) A receptor, theta	0.0409	−2.11
OR2T33	Olfactory receptor, family 2, subfamily T, member 33	0.0450	−2.03
OR5P3	Olfactory receptor, family 5, subfamily P, member 3	0.0047	−1.99
HEPH	Hephaestin	0.0393	−1.89
UNG	Uracil-DNA glycosylase	0.0280	−1.89
OAT	Ornithine aminotransferase	0.0068	−1.86
GSDMA	Gasdermin A	0.0393	−1.86
GREB1	Growth regulation by estrogen in breast cancer 1	0.0090	−1.86
DPF3	D4, zinc and double PHD fingers, family 3	0.0060	−1.77
UQCRB	Ubiquinol-cytochrome c reductase binding protein	0.0019	−1.76
LDHC	Lactate dehydrogenase C	0.0002	−1.76
S100A6	S100 calcium binding protein A6	0.0243	−1.74
C2orf70	Chromosome 2 open reading frame 70	0.0041	−1.74
SPATS2	Spermatogenesis associated, serine-rich 2	0.0060	−1.74
OR52H1	Olfactory receptor, family 52, subfamily H, member 1	0.0428	−1.70
OR13C3	Olfactory receptor, family 13, subfamily C, member 3	0.0177	−1.70
CTSG	Cathepsin G	0.0151	−1.69
SLC22A16	Solute carrier family 22, member 16	0.0405	−1.69
SDHD	Succinate dehydrogenase complex, subunit D, integral membrane protein	0.0132	−1.68
CCDC172	Coiled-coil domain containing 172	0.0002	−1.67
NTF4	Neurotrophin 4	0.0135	−1.66
OR2F1	Olfactory receptor, family 2, subfamily F, member 1	0.0274	−1.66
PGAM2	Phosphoglycerate mutase 2 (muscle)	0.0279	−1.65

Gene	Gene name	*p* value	FC
SLITRK4	SLIT and NTRK-like family, member 4	0.0007	−1.64
OR8B3	Olfactory receptor, family 8, subfamily B, member 3	0.0490	−1.64
LOC101105206	Olfactory receptor 12-like	0.0133	−1.63
C10orf53	Chromosome 10 open reading frame 53	0.0431	−1.62
FOLR1	Folate receptor 1 (adult)	0.0173	−1.61
SOHLH2	Spermatogenesis and oogenesis specific basic helix-loop-helix 2	0.0128	−1.61
ZC3H14	Zinc finger CCCH-type containing 14	0.0001	−1.59
CETN1	Centrin, EF-hand protein, 1	0.0119	−1.59
OR5H1	Olfactory receptor, family 5, subfamily H, member 1	0.0051	−1.58
C12orf42	Chromosome 12 open reading frame 42	0.0428	−1.58
GUCA1C	Guanylate cyclase activator 1C	0.0062	−1.58
PKIB	Protein kinase (cAMP-dependent, catalytic) inhibitor beta	0.0362	−1.58
OR5T1	Olfactory receptor, family 5, subfamily T, member 1	0.0106	−1.57
SYBU	Syntabulin (syntaxin-interacting)	0.0380	−1.56
FTL	Ferritin, light polypeptide	0.0439	−1.55
OR5H2	Olfactory receptor, family 5, subfamily H, member 2	0.0241	−1.55
CDA	Cytidine deaminase	0.0054	−1.55
NKAIN2	Na+/K+ transporting ATPase interacting 2	0.0020	−1.53
DMD	Dystrophin	0.0377	−1.53
OR52D1	Olfactory receptor, family 52, subfamily D, member 1	0.0149	−1.53
DACH2	Dachshund family transcription factor 2	0.0304	−1.53
DSG1	Desmoglein 1	0.0191	−1.52
MAG	Myelin associated glycoprotein	0.0401	−1.52
C9orf135	Chromosome 9 open reading frame 135	0.0242	−1.52
C14orf79	Chromosome 14 open reading frame 79	0.0392	−1.52
LRRC48	Leucine rich repeat containing 48	0.0468	−1.52
RAB44	RAB44, member RAS oncogene family	0.0432	−1.52
CRYGA	Crystallin, gamma A	0.0100	−1.51
EPB42	Erythrocyte membrane protein band 4.2	0.0425	−1.51
PDCL2	Phosducin-like 2	0.0030	−1.51
OR8S1	Olfactory receptor, family 8, subfamily S, member 1	0.0146	−1.51

Genes with fold change (FC) ≥1.5 fold and adjusted *p* value of ≤0.05.

**Table 5:** Top diseases and disorders Bio-Functions at D10 and D50 identified by IPA.

D10 vs. C	p value
Gastrointestinal disease	5.06E-04–4.62E-02
CYP3A5, IL1RN, LRRC15, ADM, HNF4A, MOGAT2, MOGAT3, HTR3A, HLA-DQA1	
Organismal injury and abnormalities	5.06E-04–4.57E-02
CYP3A5, IL1RN, LRRC15, ADM, HTR3A, NEFL, FKBP14, LIPN, SEPT5, RASGRF2, MAG	
Neurological disease	2.55E-03–4.96E-02
ADM, HTR3A, IL1RN, ATCAY, NEFL, FKBP14, SEPT5, ZBTB16, MAG, RPL10, TRMT2A, HNF4A, BRINP1, HLA-DQA1, IER3, RASGRF2	

D50 vs. C	*p* value
Neurological disease	1.74E-04–4.07E-02
MAG, MAL, FTL, FOLR1, TGIF1, HMGB1, HEPH, NTF4	
Inflammatory response	3.24E-04–4.77E-02
CAMP, HMGB1, CTSG, LTF, DMD	
Connective tissue disorders	1.97E-03–4.14E-02
CAMP, HMGB1, CTSG, LTF, FLT, TGIF1, CDA, CDH26, GPR123, NTF4	
Immunological disease	1.97E-03–3.40E-02
CAMP, CTSG, HMGB1, LTF, UNG, LYPLA2, DSG1, NTF4, S100A6, TMEM116, CDA, GPR123, CDH26	
Inflammatory disease	1.97E-03–4.46E-02
FOLR1, CAMP, CTSG, HMGB1, LTF, FTL, DMD, NKAIN2, VNN2, CDA, GPR123, CDH26, NTF4	

**Table 6:** Comparison of D10 and D50 fold-change data from microarrays and RT-qPCR.

Gene	D10				D50			
	Microarray		RT-qPCR		Microarray		RT-qPCR	
	FC	*p* value	FC	*p* value	FC	*p* value	FC	*p* value
ADM	2.3	0.0482	1.34	0.232	−1.05	0.8915	1.32	0.244
CDH26	−1.54	0.2594	−1.59	0.1593	−2.44	0.0418	−1.77	0.154
HMGB1	−1.29	0.1249	1.53	0.712	1.50	0.0279	2.2	0.403
IFI6	2.62	0.0422	2.19	0.14	1.54	0.2912	2.81	0.055
IL1RN	1.52	0.0337	−1.66	0.22	−1.05	0.7716	−1.26	0.068
MOXD1	−1.67	0.3023	−1.76	0.29	−4.38	0.0178	−2.27	0.063
TGIF	−1.04	0.8856	−1.68	0.013	−2.18	0.0310	1.13	0.376

## Appendix A Supplementary data

Fig. S1 Heat map of differentially-expressed genes at (A) D10, and (B) D50. Three infected vs two uninfected controls. All genes identified as ≥1.5 fold and *q *≤ 0.05. Each column represents a gene and each row an animal. The relative levels of expression are represented by the intensity of colour; red, increased expression and blue decreased expression within each comparison.
