# Supplementary figures and images for: The effect of PrPSc accumulation on inflammatory gene expression within sheep peripheral lymphoid tissue
Source: Vet Microbiol. 2015 Dec 31;181(3-4):204–11. doi: 10.1016/j.vetmic.2015.10.013 (PMC4678288; doi:10.1016/j.vetmic.2015.10.013)

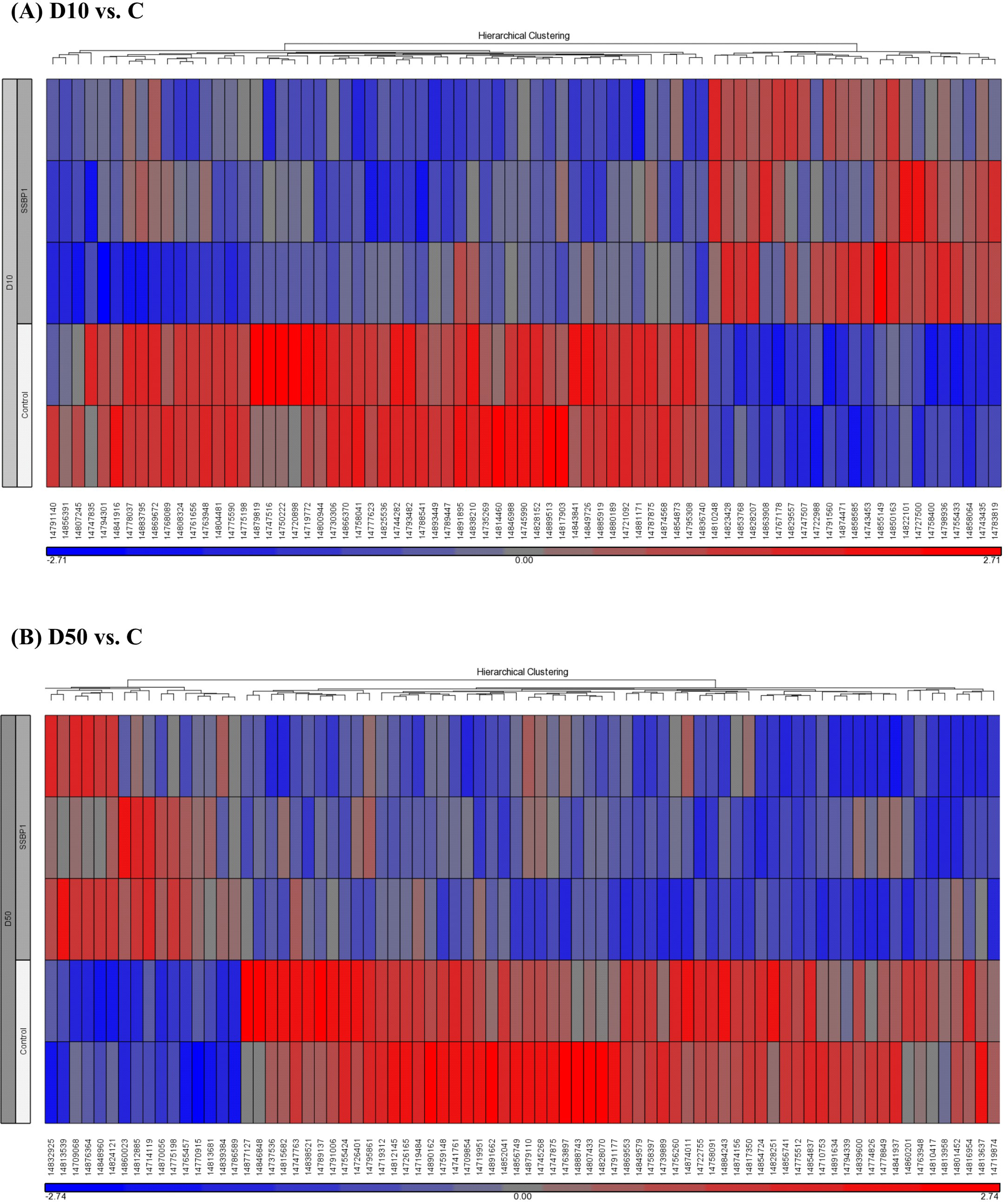

Supplement: Supplementary file 5 — Fig. S1 Heat map of differentially-expressed genes at (A) D10, and (B) D50. Three infected vs two uninfected controls. All genes identified as ≥1.5 fold and q ≤ 0.05. Each column represents a gene and each row an animal. The relative levels of expression are represented by the intensity of colour; red, increased expression and blue decreased expression within each comparison. [file mmc5.jpg]
